# Connective tissue growth factor as a novel therapeutic target in high grade serous ovarian cancer

**DOI:** 10.18632/oncotarget.6082

**Published:** 2015-11-11

**Authors:** Kim Moran-Jones, Brian S. Gloss, Rajmohan Murali, David K. Chang, Emily K. Colvin, Marc D. Jones, Samuel Yuen, Viive M. Howell, Laura M. Brown, Carol W. Wong, Suzanne M. Spong, Christopher J. Scarlett, Neville F. Hacker, Sue Ghosh, Samuel C. Mok, Michael J. Birrer, Goli Samimi

**Affiliations:** ^1^ Kinghorn Cancer Centre and Garvan Institute of Medical Research, Cancer Research Program, Darlinghurst, NSW, Australia; ^2^ St. Vincent's Clinical School, Faculty of Medicine, University of New South Wales, Sydney, NSW, Australia; ^3^ Department of Pathology and The Human Oncology and Pathogenesis Program, Memorial Sloan-Kettering Cancer Center, New York, NY, USA; ^4^ Kolling Institute of Medical Research, Royal North Shore Hospital, University of Sydney, Sydney, NSW, Australia; ^5^ FibroGen Inc., San Francisco, CA, USA; ^6^ School of Environmental & Life Sciences, University of Newcastle, Ourimbah, NSW, Australia; ^7^ School of Women's and Children's Health, University of New South Wales, and Gynaecological Cancer Centre, Royal Hospital for Women, Sydney, NSW, Australia; ^8^ Laboratory of Gynecologic Oncology, Brigham and Women's Hospital, Harvard Medical School, Boston, MA, USA; ^9^ Department of Gynecologic Oncology and Reproductive Medicine, University of Texas MD Anderson Cancer Center, Houston, TX, USA; ^10^ Harvard Medical School, Massachusetts General Hospital Cancer Center, Boston, MA, USA

**Keywords:** CTGF, FG-3019, metastasis, ovarian cancer, tumor microenvironment

## Abstract

Ovarian cancer is the most common cause of death among women with gynecologic cancer. We examined molecular profiles of fibroblasts from normal ovary and high-grade serous ovarian tumors to identify novel therapeutic targets involved in tumor progression. We identified 2,300 genes that are significantly differentially expressed in tumor-associated fibroblasts. Fibroblast expression of one of these genes, connective tissue growth factor (CTGF), was confirmed by immunohistochemistry. CTGF protein expression in ovarian tumor fibroblasts significantly correlated with gene expression levels. CTGF is a secreted component of the tumor microenvironment and is being pursued as a therapeutic target in pancreatic cancer. We examined its effect in *in vitro* and *ex vivo* ovarian cancer models, and examined associations between CTGF expression and clinico-pathologic characteristics in patients. CTGF promotes migration and peritoneal adhesion of ovarian cancer cells. These effects are abrogated by FG-3019, a human monoclonal antibody against CTGF, currently under clinical investigation as a therapeutic agent. Immunohistochemical analyses of high-grade serous ovarian tumors reveal that the highest level of tumor stromal CTGF expression was correlated with the poorest prognosis. Our findings identify CTGF as a promoter of peritoneal adhesion, likely to mediate metastasis, and a potential therapeutic target in high-grade serous ovarian cancer. These results warrant further studies into the therapeutic efficacy of FG-3019 in high-grade serous ovarian cancer.

## INTRODUCTION

Ovarian cancer is the most common cause of death among women with gynecologic cancer, responsible for 5% of cancer-related deaths [1]. The high mortality rate results from the lack of an adequate screening test for early disease, coupled with rapid progression to chemo-resistance. Although patients diagnosed with early stage disease have a 5-year survival rate of 80%, the majority of women are diagnosed at late stage, when the disease has already metastasized to multiple organs within the peritoneal cavity, resulting in a reduced 5-year survival rate of < 30% [2].

Recent molecular investigations, such as those performed by The Cancer Genome Atlas [3], have led to an increased understanding of the pathogenesis of ovarian cancer. Epithelial ovarian tumors are generally classified as serous, endometrioid, clear cell or mucinous histotypes. Serous ovarian tumors are most common, and are classified as low-grade (Type I) or high-grade (Type II) tumors [4, 5]. High grade serous ovarian cancers (HGSOC) are thought to be *de novo* invasive and are genomically unstable [4, 6], contributing to the eventual development of chemo-resistant disease in 75% of treated women [7]. As a consequence, the stromal components of the tumor, which are relatively genomically stable and essential for progression and metastasis [8], have been increasingly targeted by newly developed anti-cancer therapies [9].

Previously, studies have sought to characterize over-expression of specific ovarian tumor stromal genes in a compartmentalized fashion. These studies have demonstrated that genes such as osteonectin [10], keratinocyte growth factor [11], transforming growth factor alpha [12] and beta [13], hepatocyte growth factor [14] and kit ligand [11] are differentially expressed between normal ovary and ovarian tumor stroma. More recent studies have undertaken analyses of stromal gene expression using molecular profiling studies of laser capture microdissected stroma from HGSOC tumors [15, 16]; however, samples sizes in these studies were limited.

In this study, we perform a comprehensive molecular profiling analysis of stromal fibroblasts in 10 normal ovary samples and 51 HGSOC tumors. Furthermore, we examine the functional role of connective tissue growth factor (CTGF) in *in vitro* and *ex vivo* models of HGSOC. CTGF is a secreted stromal factor that is well established in driving extracellular matrix formation as well as proliferation, cell migration, angiogenesis and epithelial-to-mesenchymal transformation, and which has been previously identified as over-expressed in a number of other cancer types [17–20]. We show that CTGF promotes migration and peritoneal adhesion of HGSOC cells, and inhibition of CTGF by a therapeutic antibody FG-3019 abrogates these effects. Our results establish that otherwise normal fibroblasts undergo genome-wide expression changes in response to the epithelial ovarian tumor, and identify CTGF as a new potential therapeutic target in HGSOC.

## RESULTS

### Ovarian cancer-associated fibroblasts display different gene expression profiles compared to normal ovarian fibroblasts

We examined global molecular profiles for 51 ovarian tumor-associated fibroblast and 10 normal ovarian fibroblast samples. Expression of the T-cell markers CD8 and CD45 and the endothelial cell markers TIE-2 and VEGFR1 were below the level of detection in most samples, indicating that the samples were enriched for fibroblasts and not contaminated by immune or endothelial components of the stroma (Supplementary Figure S1A). Thus, fibroblasts were the major contributing component of the gene expression profiles.

**Figure 1:**
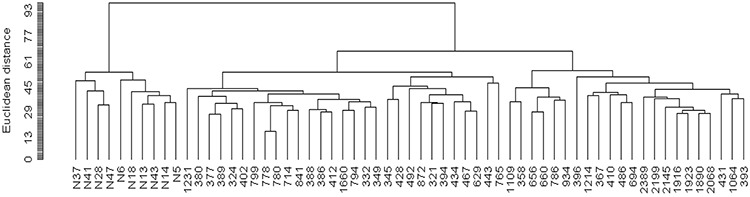
Unsupervised hierarchical clustering dendogram of microdissected fibroblasts from 51 HGSOC tumors and 10 normal ovarian tissues, using 9,741 probe sets that passed filtering criteria

Hierarchical clustering displayed two distinct branches, clearly distinguishing between normal and tumor-associated fibroblasts (Figure 1). Supervised class comparison analysis identified 2,703 probe sets, corresponding to 2,300 genes, as significantly differentially expressed between tumor-associated and normal fibroblasts (Supplementary Table S2). There was substantial overlap between our list of differentially expressed genes and those derived from 2 recent molecular profile studies of laser capture microdissected stroma from HGSOC tumors [15, 16] (Supplementary Table S3).

### Quantitative real-time PCR validation of microarray data

Nine genes differentially expressed between normal and tumor-associated fibroblasts were selected to validate the microarray results in all samples by qRT-PCR. Of the 9 genes tested, 8 (THBS1, CYR61, CTGF, MXRA5, SPP1, LTBP2, TGFBR1 and COL11A1) were found by qRT-PCR to be significantly differentially expressed in tumor-associated fibroblasts, for a validation rate of 89%. The trends in gene expression levels across normal and tumor samples were consistent between qRT-PCR and microarray analysis, with genes identified as over-expressed by microarray also found to be over-expressed by PCR (Supplementary Figure S1B).

### Connective tissue growth factor (CTGF) is over-expressed specifically in fibroblasts of HGSOC tumors

One of the genes identified as consistently up-regulated in HGSOC tumor-associated *versus* normal fibroblasts is Connective Tissue Growth Factor (CTGF). CTGF is a TGF-beta-regulated, secreted component of tumor stroma, and is well established in driving extracellular matrix formation, cell migration, angiogenesis and epithelial-to-mesenchymal transformation [21, 22]. In several cancer types, including esophageal [23], breast [24] and prostate [25], CTGF has been shown to promote tumorigenesis. Notably, CTGF is currently under clinical investigation as a viable therapeutic target in pancreatic cancer and fibrotic diseases by FibroGen Inc. (San Francisco, CA, USA). FibroGen has developed a monoclonal anti-CTGF blocking antibody FG-3019, which inhibits tumor growth and metastasis in pancreatic cancer in preclinical studies [26–28], and has been shown to be safe and well-tolerated [29]. Thus, we pursued CTGF as a therapeutic target that could be readily translated to the clinic.

**Figure 2:**
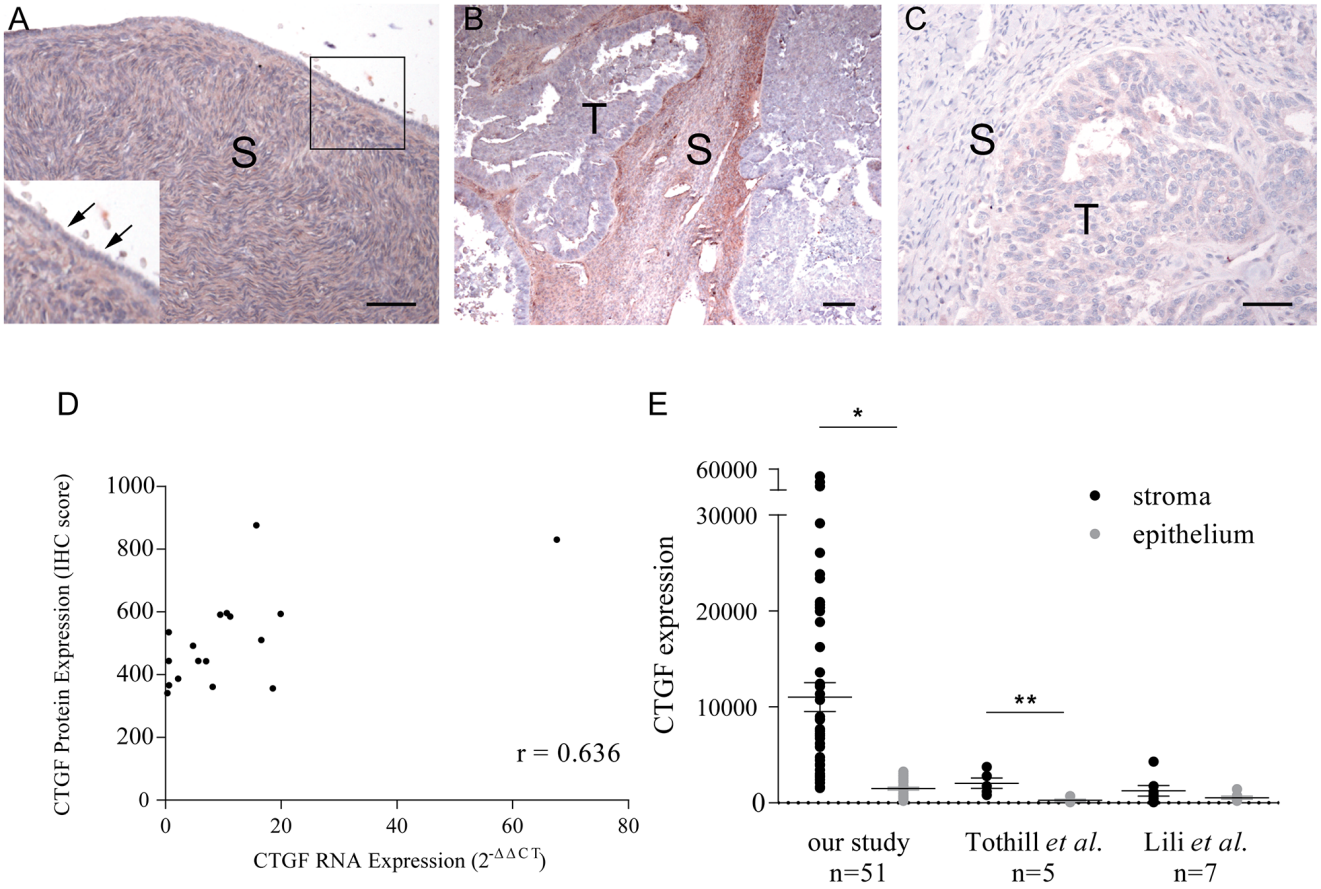
Immunohistochemical staining of CTGF on formalin-fixed tissue sections **A.** Normal ovary (inset shows higher magnification of boxed region). Arrows indicate an absence of CTGF expression in normal ovarian surface epithelium. **B.** HGSOC with high-levels of stromal CTGF expression. **C.** A negative control using normal rabbit IgG on a HGSOC with high-levels of stromal CTGF expression. S = stroma, T = tumor. Bar = 50 μm. **D.** Correlation between CTGF stromal expression by real-time PCR and by immunohistochemistry in 17 HGSOC tumors (Pearson's r = 0.636). **E.** CTGF expression in HGSOC stroma as measured by microarray in our study, and studies described by Tothill *et al*. [15] and Lili *et al*. [16]. *p-value < 10^−5^, **p-value < 0.02.

To validate CTGF expression in HGSOC tumors, immunohistochemical staining of CTGF was performed on paraffin-embedded sections from 17 HGSOC tumors and 10 normal ovarian tissues that were profiled in this study. CTGF protein expression was undetectable in the cortical stroma and surface epithelium of normal ovary (Figure 2A), while CTGF expression in HGSOC tumor stroma was significantly higher than in normal ovaries (*p*-value = 0.024) (Figure 2B). IgG staining was not detected in the tissue sections (Figure 2C). Furthermore, there was a positive correlation between CTGF gene expression in fibroblasts and IHC-derived protein expression in the stroma (*r* = 0.636) (Figure 2D). Our [30] and other gene expression data derived from matched microdissected HGSOC epithelium [15, 16] showed significantly increased CTGF expression in HGSOC stroma, compared with matched HGSOC epithelial cells (Figure 2E). These findings indicate that CTGF expression is primarily restricted to tumor-associated fibroblasts in HGSOC.

### CTGF promotes migration, anchorage-independent growth and peritoneal adhesion of HGSOC cell lines

**Figure 3:**
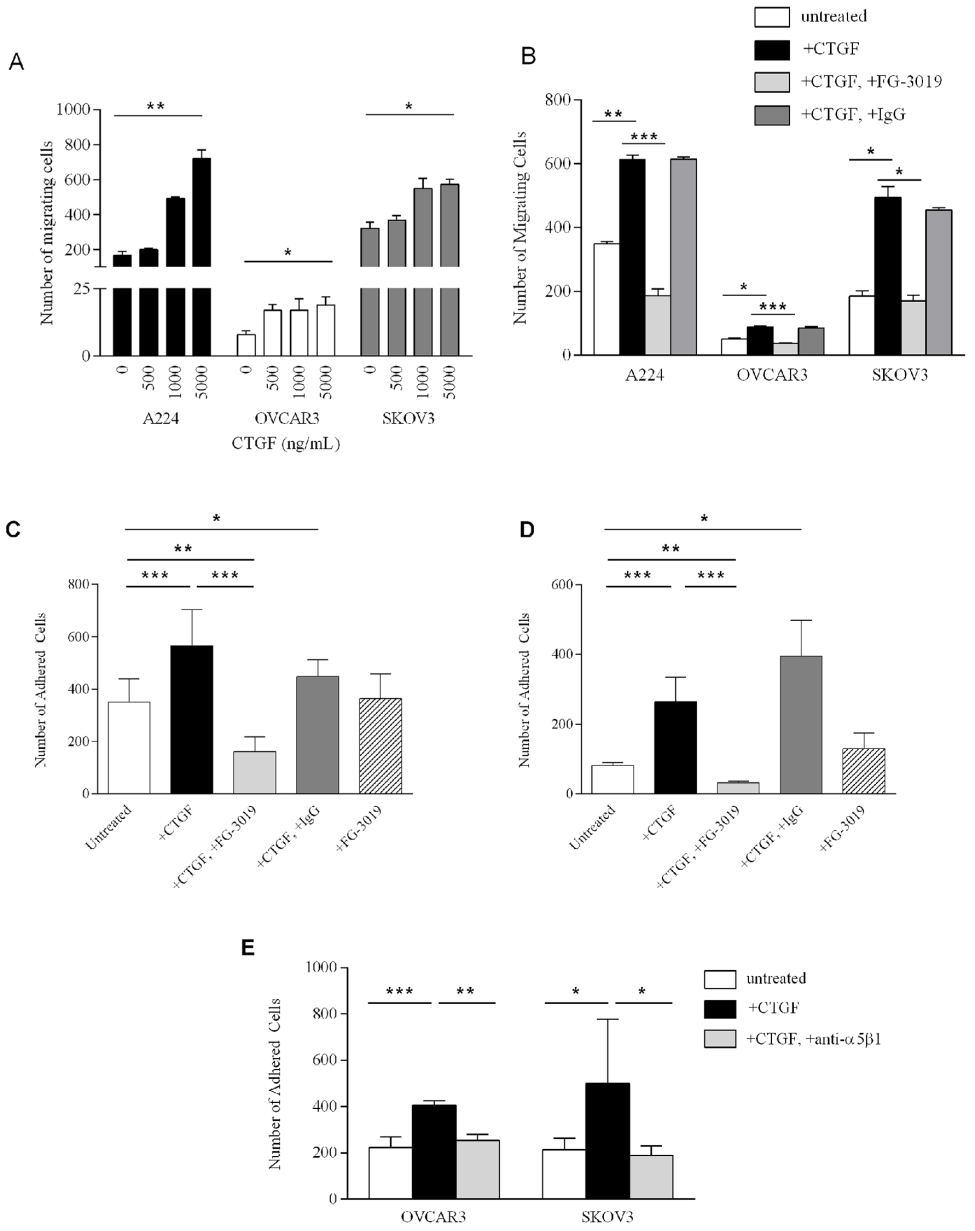
Functional studies of CTGF and FG-3019 **A.** Migration of A224, OVCAR3 and SKOV3 cells in response to increasing concentrations of rhCTGF. **B.** 6-hour migration of A224, OVCAR3 and SKOV3 cells (untreated); with 5 μg/ml CTGF; with 5 μg/ml CTGF+100 μg/ml FG-3019 and with 5 μg/ml CTGF+100 μg/ml IgG. Each bar represents the mean of triplicate wells ± SD. *Ex vivo* peritoneal tissue adhesion of OVCAR3 cells **C.** and SKOV3 cells **D.** Untreated; with 5 μg/ml rhCTGF (CTGF) with 5 μg/ml rhCTGF+50 μg/ml FG-3019; with 5 μg/ml rhCTGF+50 μg/ml IgG and with 50 μg/ml FG-3019. Each bar represents the average adhesion in at least 2 wells in 2 independent experiments ± SD. **E.** *Ex vivo* peritoneal tissue adhesion of OVCAR3 and SKOV3 cells: untreated; with 5 μg/ml rhCTGF; and with 5 μg/ml rhCTGF/20 μg/ml anti-α5β1 antibody. Each bar represents the average adhesion in at least 2 wells in 2 independent experiments ± SD. *p-value < 0.05, **p-value < 0.01, ***p-value < 0.005.

We sought to determine the effect of exogenous CTGF, and its inhibition, on tumor-promoting processes. Migration of A224, OVCAR3 and SKOV3 ovarian cancer cells lines through transwells was significantly increased upon addition of recombinant human CTGF (rhCTGF), in a dose-dependent manner (Figure 3A). Addition of 5 μg/ml rhCTGF significantly stimulated migration of A224 (*p*-value < 0.008), OVCAR3 (*p*-value < 0.02) and SKOV3 (*p*-value < 0.02), while addition of 100 μg/ml CTGF-blocking antibody FG-3019 significantly decreased transwell migration in the presence of rhCTGF in A224 (*p*-value < 0.004), OVCAR3 (*p*-value < 0.003) and SKOV3 (*p*-value < 0.02) (Figure 3B).

Anchorage-dependent (plastic) and anchorage-independent (soft agar) cell proliferation was measured in A224, OVCAR3 and SKOV3 cells. Addition of 5 μg/ml rhCTGF in anchorage-dependent cell proliferation assays did not promote proliferation over a 4-day period (Supplementary Figure S2A). For measurement of anchorage-independent growth, we established CTGF-over-expressing clones from OVCAR3 cells to stably express and secrete CTGF. Because of the long incubation times required for soft agar assays, this approach was necessary to overcome the instability of rhCTGF in culture media. All three CTGF-over-expressing clones demonstrated significantly increased anchorage-independent growth compared to empty vector controls (*p* < 0.0001) (Supplementary Figure S2B). These findings are consistent with previous studies, which demonstrated that CTGF expression enhanced anchorage-independent growth in the MIA PaCa-2 pancreatic cancer cell line, while having no effect on monolayer growth [29]. Peritoneal metastasis is common in ovarian cancer and represents a major challenge in treatment. During tumor progression, HGSOC cells from the ovary disseminate throughout the peritoneal cavity and adhere to the peritoneal wall [31, 32]. CTGF expression has been examined in human peritoneal tissue, and shown to be present in both the mesothelial cells and the peritoneal fibroblasts [33]. IHC on mouse peritoneal tissue similarly reveals CTGF expression within the mesothelial layer, and fibroblasts (Supplementary Figure S3). To determine whether CTGF mediates peritoneal adhesion, we developed an *ex vivo* peritoneal adhesion assay and measured adhesion of cells to mouse peritoneal tissue. Addition of 5 μg/ml rhCTGF significantly increased adhesion of OVCAR3 (*p*-value < 0.002) and SKOV3 (*p*-value < 0.001) cells to peritoneal tissue (Figure 3C and 3D, respectively). Addition of 50 μg/ml FG-3019 significantly inhibited rhCTGF-mediated peritoneal adhesion in OVCAR3 (*p*-value < 6 × 10^−5^) and SKOV3 (*p*-value < 0.0003) (Figure 3C and 3D, respectively). Interestingly, adhesion in the rhCTGF+FG-3019-treated cells was significantly decreased compared to untreated cells (*p*-value < 0.006 for OVCAR3, *p*-value < 0.002 for SKOV3), suggesting that FG-3019 may be acting upon endogenous CTGF expressed in the peritoneal tissue.

CTGF has been shown to bind to the fibronectin receptor integrin α5β1 and promote migration in pancreatic cells [34], and integrin α5β1 has been demonstrated to mediate peritoneal adhesion of ovarian cancer cells [35]. To investigate whether CTGF mediates its effect on HGSOC cells via α5β1, we examined the effect of blocking the activity of integrin α5β1 on peritoneal adhesion of OVCAR3 and SKOV3 cells (Figure 3E). rhCTGF-stimulated peritoneal adhesion was significantly hindered upon treatment with anti-integrin α5β1 antibody compared to rhCTGF-treated cells (*p-value* < 0.008 for OVCAR3, *p*-value < 0.03 for SKOV3). Treatment of HGSOC cells with anti-integrin α5β1 alone had no effect on peritoneal adhesion (data not shown).

### Association between CTGF expression and clinico-pathologic characteristics

**Figure 4:**
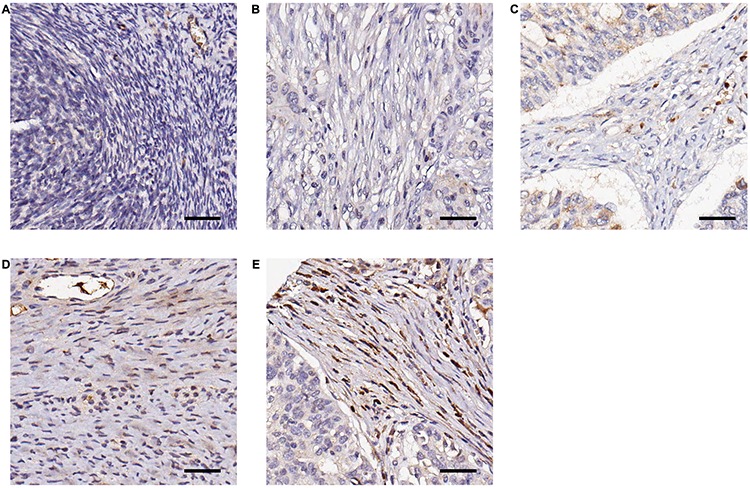
Representative examples of immunohistochemical staining of CTGF by FG-3145 in stromal fibroblasts of formalin-fixed HGSOC tissues **A.** Normal ovary; no CTGF expression **B.** tumor; no CTGF expression **C.** tumor; intensity = 1, percentage = 30% **D.** tumor; intensity = 2, percentage = 70% **E.** tumor; intensity = 3, percentage = 95%. Bar = 100 μm.

Our studies indicate that CTGF may serve as a novel therapeutic target in HGSOC by promoting steps within the metastatic process (motility and adhesion), which are inhibited by treatment with FG-3019. We wished to further characterize CTGF as a prognostic factor in an independent cohort of HGSOC cases, and to identify those patients who may be more likely to respond to treatment with FG-3019. We performed a retrospective study to examine CTGF expression in 93 HGSOC primary tumors and 10 normal ovarian tissue controls, and correlated staining with clinico-pathologic characteristics including overall survival (Figure 4, Table 1). CTGF staining was significantly higher in HGSOC samples compared to normal controls (*p*-value = 0.013). CTGF expression was not significantly associated with stage or grade of disease. Increasing percentage of stromal cells expressing CTGF was directly correlated with poorer overall survival (Supplementary Figure S4). The greatest separation in survival was between patients with ≤ 90% of cells *versus* those with > 90% of cells expressing CTGF (*p*-value = 0.006). These patients did not differ from the rest of the cohort with respect to age, stage/grade or debulking/cytoreduction status. However, the number of cases in this category was limited (*N* = 4), and additional studies with larger sample sizes will be necessary to verify this association. In agreement with this trend of increasing CTGF protein expression associated with decreased survival, we identified a similar association between CTGF gene expression and survival in the TCGA dataset [3], with those cases expressing highest levels of CTGF (*z*-score > 2, *N* = 15) having the poorest survival (Supplementary Figure S5).

**Table 1:** Clinical characteristics of the HGSOC cohort examined in this study

Variables	Total Cohort		
	*N* = 93 (%)	Median OS (months)	*p*-value (Log-rank)
**Age (years)**			
Mean	60.9		
Median	60.0		
Range	40.0–86.0		
**Outcome**			
Follow-up (months)	4.0–183.0		
Median follow-up (months)	87.0		
Death from ovarian cancer	64 (68.8)		
Death from other	1 (1.1)		
Death from unknown	4 (4.3)		
Alive	24 (25.8)		
**Stage (*N* = 92)**			
I	5 (5.4)	N/A	
II	4 (4.3)	81.0	
III	69 (75.0)	38.0	
IV	14 (15.2)	15.0	0.0060^a^
**Grade**			
2	30 (32.3)	44.0	
3	63 (67.7)	32.0	0.1443
**Residual disease (*N* = 60)**			
0	19 (31.7)	92.0	
> 0 to 1	24 (40.0)	38.0	
> 1 to 2	5 (8.3)	21.0	
> 2	12 (20.0)	15.0	< 0.0001
**Complete response**			
Yes	44 (47.3)	44.0	
No	49 (52.7)	28.0	0.0459
**Menopause (*N* = 92)**			
Pre-menopause	8 (8.7)	38.0	
Peri-menopause	5 (5.4)	32.0	
Post-menoapuse	79 (85.9)	35.0	0.9590
**CTGF expression (%) (*N* = 88)**			
≤ 90	84 (95.5)	38.0	
> 90	4 (4.5)	9.0	0.0006

HGSOC = high-grade serous ovarian cancer; OS = overall survival; N/A = not applicable, as median survival has not been reached; CTGF = connective tissue growth factor.

a Any CTGF expression *versus* no expression

## DISCUSSION

Tumor stroma plays a crucial role in promoting transformation and progression of cancer cells, making stromal factors attractive targets for chemo-prevention and chemotherapy intervention, and studies are investigating this novel approach (reviewed in [36]). While numerous studies have investigated CTGF as a stromal target in various diseases, to date these studies of CTGF expression and action in ovarian cancer have reported conflicting findings. Gery *et al*. first demonstrated that CTGF is over-expressed in epithelial ovarian tumors, and that expression correlates with stage of disease [37], similar to our studies showing that highest stromal CTGF expression in a subset of HGSOC was associated with a poor outcome. Tothill *et al*. described a molecular signature in a subset of HGSOC samples that was primarily driven by a high stromal response [15]. Similar to our findings, the gene expression data from this study demonstrated that CTGF was over-expressed in the stroma of these tumors, and expression was also associated with a poor outcome, suggesting that CTGF expression may promote aggressive disease [15]. However, a recent immunohistochemical analysis of CTGF expression in 107 invasive ovarian carcinomas found that loss of CTGF was associated with a poor prognosis [38], although it is unclear whether stromal expression was examined. While functional and genomic studies clearly indicate CTGF as a novel therapeutic target, it remains to be determined whether CTGF expression alone is prognostic. Additional investigations in stratified studies are necessary to identify the subset(s) of patients in which CTGF expression may be used as a prognostic biomarker.

Our studies and others suggest that CTGF inhibition by FG-3019 may have beneficial effects, as tumors with increased stromal involvement present with poorer survival. Our studies demonstrate that FG-3019 treatment decreases CTGF-induced HGSOC migration and peritoneal adhesion *in vitro*. Additional studies have indicated that targeting CTGF may be most beneficial in desmoplastic, chemotherapy-resistant tumors, which are difficult to model in ovarian cancer with existing systems. Neesse *et al*. [28] investigated whether inhibition of the tumor stromal compartment in pancreatic ductal adenocarcinoma could improve survival *in vivo*. Inhibition of CTGF by FG-3019 treatment in mice carrying gemcitabine-resistant tumors resulted in increased tumor apoptosis, response and survival [28]. Future studies would address this hypothesis by examining the effect of CTGF inhibition on cisplatin-resistant tumor growth and survival; we are currently preparing for studies to investigate FG-3019 efficacy in cisplatin-resistant HGSOC patient-derived xenografts.

CTGF expression and effect are likely to be context-dependent, and different cell/tumor types respond differently to CTGF. For examples, studies have reported that CTGF expression is down-regulated in *in vitro* 3D models of epithelial ovarian cancer [39, 40], while another report described increased CTGF expression in ovarian cancer cell lines capable of spheroid formation [41]. A study using epithelial cell lines derived from endometrioid, clear cell, and mucinous histotypes of ovarian cancer, all molecularly and clinically distinct from HGSOC, showed methylation-driven inactivation of CTGF. Over-expression of CTGF in these cell lines subsequently resulted in decreased growth [42]. It is likely then that CTGF expression and cellular effects are dependent on, or influenced by, the tumor microenvironment.

A recent study by Zhang *et al*. undertook an integrative genomic, epigenomic and transcriptomic analysis in ovarian cancer to further elucidate the poor prognosis for ovarian cancer patients [43]. They identified several functions related to tumor progression that were enriched in subtypes with the poorest prognosis, including cell adhesion, growth factor binding, motility and angiogenesis. Notably, these functions include CTGF as an up-regulated gene in this poor prognosis subtype. Similarly, our results suggest that CTGF may promote the metastatic potential of HGSOC by increasing cellular motility and adhesion of tumor epithelial cells to peritoneal tissue, revealing CTGF as a new potential target and uncovering a novel therapeutic approach in HGSOC.

## MATERIALS AND METHODS

### Tissue specimens

Primary HGSOC tumors were obtained as described [44] from previously untreated ovarian cancer patients hospitalized at the Brigham and Women's Hospital between 1990 and 2000. Classification was determined according to the International Federation of Gynecology and Obstetrics standards. Normal ovaries were obtained from patients who had undergone surgery for benign gynecologic diseases. The Garvan Institute of Medical Research Tissue Microarrays were constructed from formalin-fixed, paraffin-embedded tissue specimens from women undergoing primary laparatomy at the Gynaecological Cancer Centre, Royal Hospital for Women, Sydney, between 1989 and 2002. Surgical, clinical and histopathologic data (histopathologic diagnosis, FIGO stage, surgical debulking, tumor grade, survival) were extracted from medical records. All specimens and their corresponding clinical information were collected by written consent under protocols approved by the institutional review boards of the respective institutions.

### Microdissection and RNA isolation, amplification and hybridization

Microdissection and RNA isolation were performed as previously described [44]. Briefly, fibroblasts were identified by a pathologist and microdissected from 7 μm frozen sections of ovarian tumors or normal ovary using a MD LMD laser microdissecting microscope (Leica, Wetzlar, Germany). RNA was isolated immediately in RLT lysis buffer and was extracted and purified using the RNeasy Micro kit (Qiagen, Valencia, CA). All purified total RNA specimens were quantified and checked for quality with a Bioanalyzer 2100 system (Agilent, Palo Alto, CA). Total RNA amplification and hybridization were performed as previously described [44]. (GEO accession number GSE40595).

### Data normalization, filtering and analysis

Global normalization, quality control screening and collation were performed as previously described [44]. Normalized data were uploaded into the NCI Microarray Analysis Database for quality-control screening and collation. BRB ArrayTools (version 3.5.0) software developed by Dr. Richard Simon and Amy Peng Lam (National Cancer Institute, Bethesda, MD) was used to filter the array data, selecting only those probe sets that were present in > 50% of the arrays and whose expression was varied among the top 50th percentile, and complete the statistical analysis. The filtered data set (9,741 probe sets) was used for hierarchical clustering, using a Euclidean distance metric with average linkage, and class comparison analysis between tumor-associated and normal fibroblasts. The resulting gene list (2,703 probe sets) contained < 10 false positives at a confidence of 95%. Differential expression was considered significant at *p*-value < 0.001.

### Quantitative real-time PCR

Quantitative real-time PCR (qRT-PCR) was performed on 100 ng of double-amplified product from all specimens using primer sets specific for 9 genes (selected either at random or as a gene of interest), as well as 2 normalizing genes, beta-glucuronidase (GUSB) and cyclophilin (Supplementary Table S1). An iCycler iQ Real-time PCR Detection System (Bio-Rad Laboratories, Hercules, CA) was used in conjunction with the SuperScript III Platinum SYBR Green One-Step qRT-PCR kit (Invitrogen, Carlsbad, CA) according to the manufacturer's instructions.

### Immunohistochemistry of tissue sections

Immunolocalization of CTGF protein was performed on formalin-fixed paraffin-embedded tissue sections using a commercially available anti-CTGF polyclonal antibody (ab6992, Abcam, Cambridge, UK) and the Picture MAX system (Zymed Laboratories Inc, Carlsbad, CA). Samples were de-paraffinized by incubating in xylene, rehydrated by soaking in 95% ethanol, followed by antigen retrieval in Target Retrieval Solution (DAKO, Carpinteria, CA) at 120°C for 20 minutes. Slides were treated for endogenous peroxidase activity in 3% hydrogen peroxide and sections were incubated with primary antibody (1:50 dilution) at room temperature for 60 minutes, washed twice with 1x TBS and incubated with HRP polymer for 30 minutes. CTGF-positive signals were visualized using ACE Single Solution (Zymed Laboratories Inc, Carlsbad, CA). As negative control, normal rabbit IgG was applied to the HGSOC with high-levels of stromal CTGF expression. Stromal CTGF protein expression was quantified in one or two sections per case using Image-Pro Plus 5.1.0.20 for Windows (Media Cybernetics, Bethesda, MD), as previously described [45]. The staining saturation was measured from 5 fixed-size areas in the stroma of both tumor and normal ovaries and averaged, yielding one score for each case.

### Tissue culture

A224, OVCAR3 and SKOV3 ovarian cancer cell lines were cultured in RPMI medium (Invitrogen, Carlsbad, CA) supplied with 10% fetal bovine serum and 20 mM L-glutamine and maintained in a humidified incubator at 37° and 5% CO_2_. Cell lines were authenticated by STR analysis.

### Materials

Recombinant human CTGF (rhCTGF), purified from a stable CTGF over-expressing CHO cell line, FG-3145 and FG-3019 were provided by FibroGen, Inc. (San Francisco, CA). FG-3019 is a fully human IgG1 mAb recognizing domain 2 of human and rodent CTGF. FG-3145 is a mouse mAb against human CTGF, used for immunohistochemistry studies. Normal mouse IgG (Santa Cruz Biotech, Santa Cruz, CA) was used as a control.

### Cell migration assays

Cell motility was determined using 8 micron PET membrane transwell culture chambers (BD Biosciences, San Jose, CA). Cells were serum-starved overnight. RPMI media/10% serum (500 μl) was added to lower wells and cells were seeded in 350 μl serum-free RPMI media in the upper wells. Recombinant CTGF (5 μg/ml), FG-3019 (100 μg/ml) and/or IgG (100 μg/ml) were added to the top and bottom wells, and the chambers were incubated at 37°C for 6 hours. The non-motile cells were removed from the upper surface of the membrane with a cotton-tipped swab. The membranes were then fixed and stained using Diff-Quik stain (Dade Behring, Deerfield, IL). Three independent experiments were performed with triplicate samples. The number of migrating cells was calculated by counting the total number of cells in 5 fields at 20X magnification.

### *Ex vivo* peritoneal assays

Ovarian cancer cell peritoneal adhesion was determined using an *ex vivo* assay, modified from previous studies [46]. Briefly, the peritoneal tissue was excised from euthanized 10–12 weeks-old female Balb/c mice, divided along the midline into two pieces and placed into serum-free media. In 96-well plates, 100 μL of medium containing 5 × 10^4^ Syto9-labeled cells was added to 100 μL of medium containing rhCTGF (final concentration 5 μg/mL), FG-3019 (final concentration 50 μg/mL), or IgG (final concentration 50 μg/mL). Anti-integrin α5β1 antibody (MAB1969, clone JBS5, Chemicon) was used at a final concentration of 20 μg/ml. Peritoneal tissue (mesothelial surface facing downward) was laid over the wells and was covered by a glass coverslip and the plate lid. The plate was incubated upside-down for 2 hours at 37°C. The peritoneal tissue was then washed with serum-free medium, and attached cells observed and imaged using a Leica MZ16FA fluorescent dissection microscope, attached to a Leica DFC420C camera. Image J was used to count 3 fields per well.

### Immunohistochemistry of tissue microarrays

Garvan Institute tissue microarrays were constructed as previously described [47]. Tissue microarray sections were de-paraffinized by incubation in xylene, followed by antigen retrieval using DAKO pH9 solution (DAKO, Carpinteria, CA) for 4 minutes. Endogenous activity was then blocked with 3% H_2_0_2_. Tissue microarrays were incubated with anti-CTGF antibody FG-3145 (FibroGen Inc., San Francisco, CA) at 30 μg/mL for 60 minutes. The Novocastra Novolink Polymer Detection System (Leica Biosystems, Buffalo Grove, IL) and substrate chromogen DAB was used for visualization. The percentage of stromal fibroblasts stained (0–100%) and the intensity of staining (0–3) was blindly scored by a surgical pathologist (RM).

### Statistical analysis

For gene expression by quantitative real-time PCR, relative expression was calculated using the 2^−ΔΔCT^ method, using CT values for two housekeeping genes as a single reference. The Mann-Whitney *U* Test was used to compare medians of continuous variables between two independent samples in the immunohistochemistry study. R values indicate Pearson's correlation coefficients. For the *in vitro* studies, comparisons were made using two-tailed Student's *t*-test with the assumption of unequal variance and an alpha of 0.05. For analysis of clinic-pathologic associations, median survival was estimated using the Kaplan-Meier method and the difference was tested using the Log-Rank Test. The 5-year survival rate was estimated using the life-table method. *P*-values < 0.05 were considered statistically significant.

## SUPPLEMENTARY FIGURES AND TABLES






