# Supplementary material for: Connective tissue growth factor as a novel therapeutic target in high grade serous ovarian cancer
Source: Oncotarget. 2015 Nov 11;6(42):44551–62. doi: 10.18632/oncotarget.6082 (PMC4792575; doi:10.18632/oncotarget.6082)
Supplement: Supplementary file 1 [file oncotarget-06-44551-s001.pdf]

SUPPLEMENTARY FIGURES AND TABLES

A.

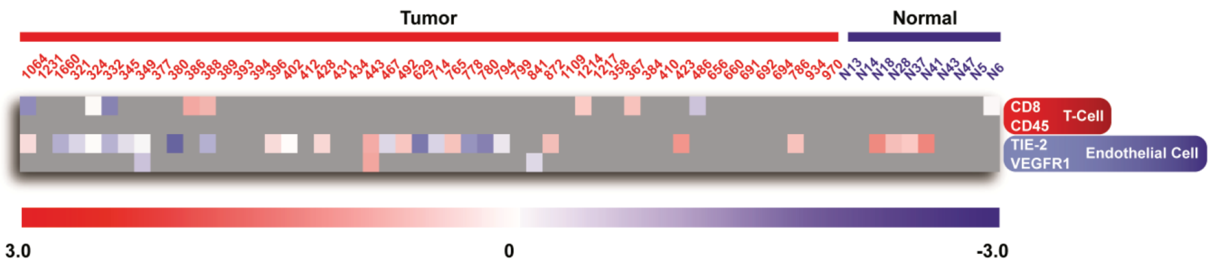

B.

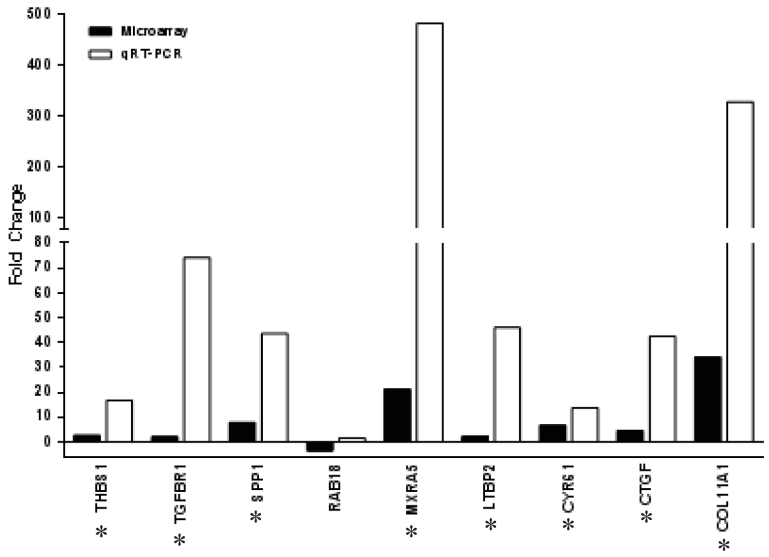

Supplementary Figure S1: A. Gene expression levels of markers of endothelial cells (TIE-2 and VEGFR1) and T-cells (CD8 and CD45) in normal ovarian and HGSOC-associated fibroblasts analyzed in this study. **B.** Validation of microarray gene expression differences by quantitative real-time PCR. qRT-PCR data were calculated using the $2^{-\Delta\Delta CT}$ method. Fold-change depicts HGSOC-associated/normal ovarian fibroblast expression. *p-value < 10^{-2}.

.

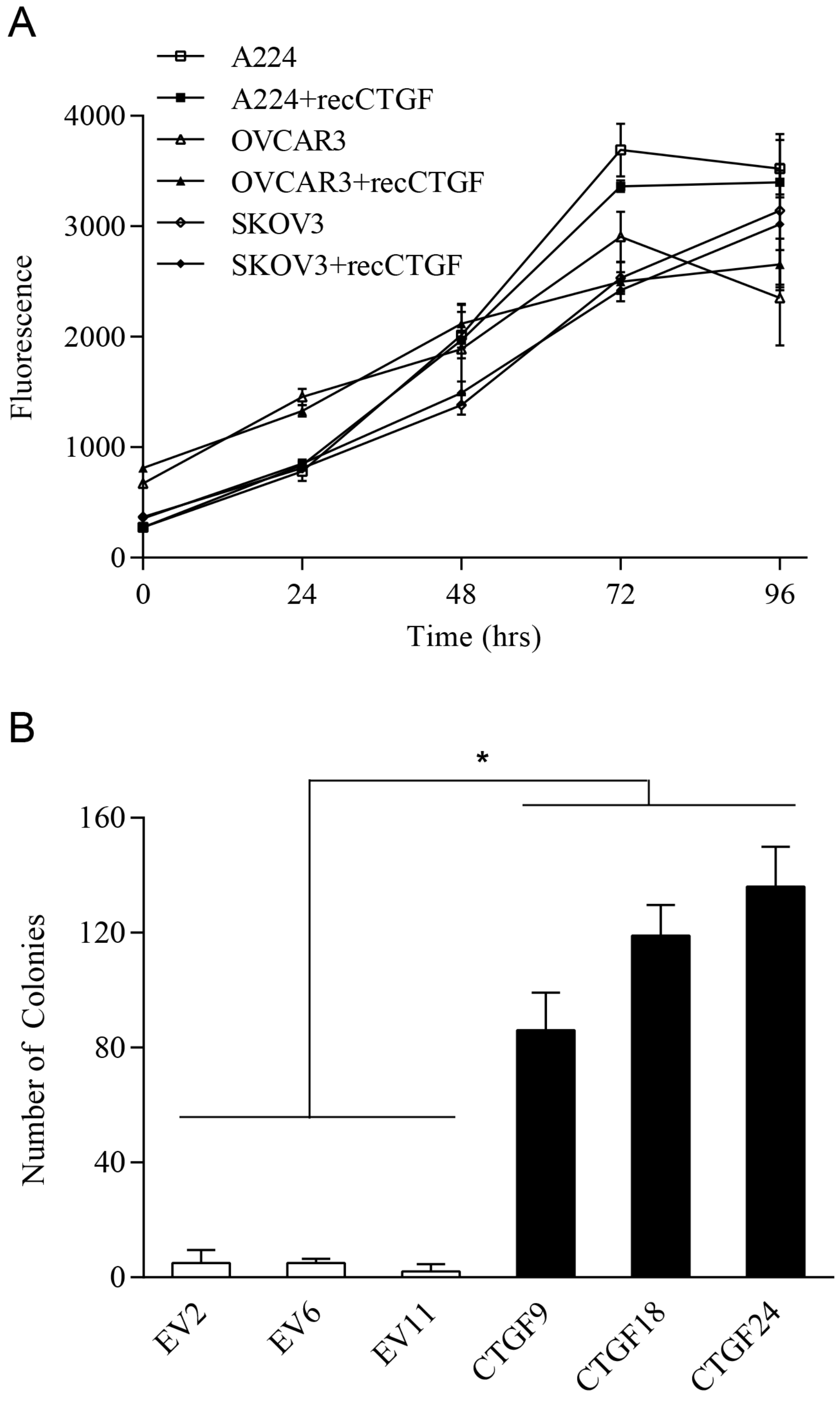

Supplementary Figure S2: A. Cell proliferation of A224, OVCAR3 and SKOV3 cells over 4 days with and without addition of 5 µg/ml recombinant CTGF. Each point represents the mean of quadruplicate wells ± SD. **B.** Anchorage-independent growth by soft agar colony formation, using OVCAR3 clones stably transfected with either empty vector (EV), or a CTGF-expressing vector (CTGF). Each bar represents the mean of triplicate wells ± SD. *p < 0.0001.

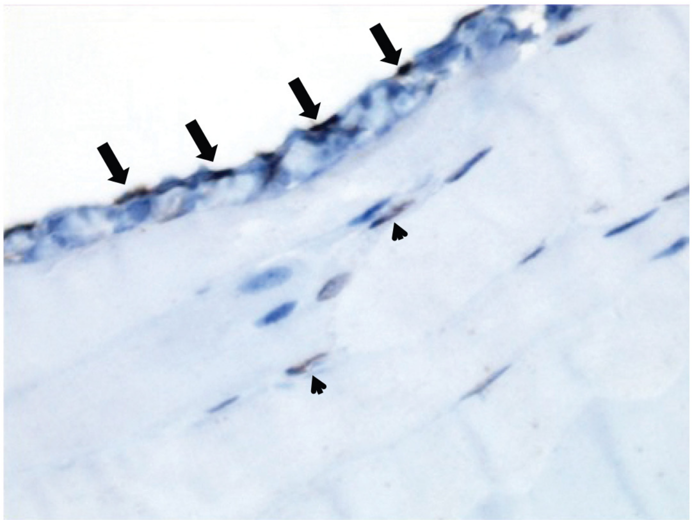

Supplementary Figure S3: CTGF is expressed in mesothelial cells (block arrows) and fibroblasts (arrowheads) in peritoneal tissue of Balb/c mice.

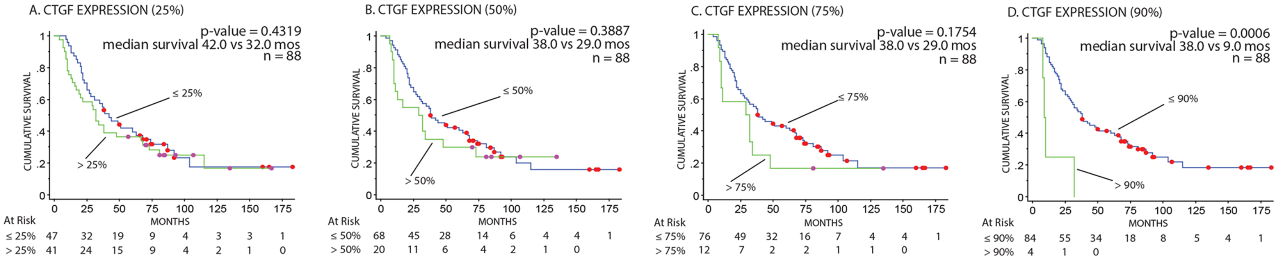

Supplementary Figure S4: Kaplan-Meier overall survival curves in HGSOC TMA cases with 25% **A.** 50% **B.** 75% C. or 90% D. of stromal cells expressing CTGF protein. P-values and differences in median survival are indicated for each survival comparison.

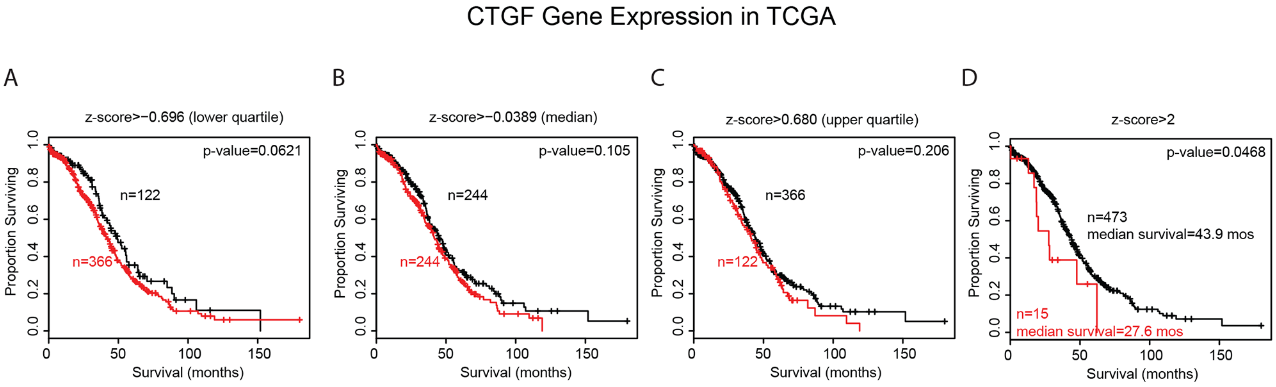

Supplementary Figure S5: Kaplan-Meier overall survival curves of the TCGA HGSOC dataset. Survival was compared in cases with CTGF gene expression below and above the lower quartile **A.** median **B.** upper quartile **C.** and in cases with high (z-score > 2) and low (z-score ≤ 2) CTGF gene expression **D.** *P*-values are indicated for each survival comparison.

Supplementary Table S1: Primer Sequences for qRT-PCR validation

Name	Sequence
GUSB-F	ACTCTTGGTATCACGACTACG
GUSB-R	CTGCTCCATACTCGCTCTG
Cyclophilin-F	TTCTTCATCACCTATGGCAAAC
Cyclophilin-R	GCAACTTCTCCAACTCATCTAG
CTGF-F	TCACTGACCTGCCTGTAG
CTGF-R	GCTGAGTCTGCTGTTCTG
CYR61-F	GCTTGTGGAGTTGATGAC
CYR61-R	TCAGGAAGGGATGGAATG
SPP1-F	CGTGGGAAGGACAGTTATG
SPP1-R	GCTCATTGCTCTCATCATTAG
TGFBR1-F	GGTATTATGTTGCTCAGTTAC
TGFBR1-R	GGCTTCTCAGTATCATTCG
THBS1-F	TTGTTGTGTGACTGAGTAAAG
THBS1-R	CCTTATTGGGAATACTTCTCTG
MXRA5-F	GTCACCACTGTTATATTACCTTCTC
MXRA5-R	CTCTCCTCTCACCTCCTCTG
LTBP2-F	CACCATCGGAGAACTTACC
LTBP2-R	CTCAAGGTTGGCAGGAAG
RAB18-F	CAAATAGGCTTGCTGTGTTTC
RAB18-R	CTCTCTTGTCTTTCTACCATCC
COL11A1-F	AAGTTGGTGTGGCTAAGATG
COL11A1-R	CCTGGATGGATGAGAATGAG

Supplementary Table S2: List of 2,703 probe sets identified as significantly differentially expressed between HGSOC-associated fibroblasts and normal ovarian fibroblasts, using a multivariate permutation test providing 95% confidence that there were no more than 10 false discoveries (*p-value* < 0.001).

Supplementary Table S3: List of 48 probe sets commonly identified as differentially expressed in HGSOC stroma.
